# Cooperative Dynamic Motion Planning for Dual Manipulator Arms Based on RRT*Smart-AD Algorithm

**DOI:** 10.3390/s23187759

**Published:** 2023-09-08

**Authors:** Houyun Long, Guang Li, Fenglin Zhou, Tengfei Chen

**Affiliations:** School of Mechanical Engineering, Hunan University of Technology, Zhuzhou 412001, China; hut_lhy@outlook.com (H.L.);

**Keywords:** RRT*Smart, dual manipulator arms, dynamic motion planning, A* cost function sampling, quintic NURBS

## Abstract

Intelligent manufacturing requires robots to adapt to increasingly complex tasks, and dual-arm cooperative operation can provide a more flexible and effective solution. Motion planning serves as a crucial foundation for dual-arm cooperative operation. The rapidly exploring random tree (RRT) algorithm based on random sampling has been widely used in high-dimensional manipulator path planning due to its probability completeness, handling of high-dimensional problems, scalability, and faster exploration speed compared with other planning methods. As a variant of RRT, the RRT*Smart algorithm introduces asymptotic optimality, improved sampling techniques, and better path optimization. However, existing research does not adequately address the cooperative motion planning requirements for dual manipulator arms in terms of sampling methods, path optimization, and dynamic adaptability. It also cannot handle dual-manipulator collaborative motion planning in dynamic scenarios. Therefore, in this paper, a novel motion planner named RRT*Smart-AD is proposed to ensure that the dual-arm robot satisfies obstacle avoidance constraints and dynamic characteristics in dynamic environments. This planner is capable of generating smooth motion trajectories that comply with differential constraints and physical collision constraints for a dual-arm robot. The proposed method includes several key components. First, a dynamic A* cost function sampling method, combined with an intelligent beacon sampling method, is introduced for sampling. A path-pruning strategy is employed to improve the computational efficiency. Strategies for dynamic region path repair and regrowth are also proposed to enhance adaptability in dynamic scenarios. Additionally, practical constraints such as maximum velocity, maximum acceleration, and collision constraints in robotic arm applications are analyzed. Particle swarm optimization (PSO) is utilized to optimize the motion trajectories by optimizing the parameters of quintic non-uniform rational B-splines (NURBSs). Static and dynamic simulation experiments verified that the RRT*Smart-AD algorithm for cooperative dynamic path planning of dual robotic arms outperformed biased RRT* and RRT*Smart. This method not only holds significant practical engineering significance for obstacle avoidance in dual-arm manipulators in intelligent factories but also provides a theoretical reference value for the path planning of other types of robots.

## 1. Introduction

As production tasks become increasingly complex, dual manipulator arm collaboration can better meet practical production needs. The development of dual manipulator arm motion planning for collaborative tasks has been a topic of significant interest.

In the existing methods for dual-arm motion planning, there are learning-based motion planning methods, intelligent optimization algorithm methods, and sample-based methods, among others. The use of reinforcement learning and deep learning in robotic arm path planning is being widely applied. Reinforcement learning [1,2] is a method of learning optimal behavior through interactions between an agent and its environment, while deep learning [3] is a machine learning technique that learns complex patterns and representations through multi-layer neural networks. However, these methods also present challenges. Training a reliable reinforcement learning model requires a lot of experimentation, iteration, and parameter tuning, which can be time-consuming. In addition, the training process of deep learning models requires a large amount of data and computational resources, making it sensitive to parameters. Therefore, suitable datasets and computing facilities are limiting factors for the application of these methods. Researchers have incorporated intelligent algorithms into robotic arm trajectory planning to optimize trajectories that meet kinematic and dynamic constraints. Traditional methods like polynomial interpolation can achieve obstacle avoidance and reasonable trajectory selection, but they lack optimal performance. To address this, genetic algorithms [4], particle swarm algorithms [5], and ant colony algorithms [6] have been commonly used to optimize robotic arm trajectory planning for superior performance. Intelligence algorithms have some drawbacks. First, they often require longer computation time due to the collaboration of multiple individuals or particles. This can lead to delays in real-time applications, limiting their performance. Second, these algorithms are highly sensitive to parameter settings, requiring extensive tuning. Their results can vary, making stability and reliability a challenge. Additionally, the random search strategy they employ can result in unstable and non-reproducible outcomes. Lastly, while effective in larger search spaces, swarm intelligence algorithms can suffer from reduced efficiency when dealing with complex problems. For sampling-based path planning methods, the A* algorithm [7] is a type of heuristic search algorithm that prioritizes selecting the optimal path by utilizing a heuristic evaluation function during the path search process. It builds a search tree to represent possible paths and selects the most promising nodes for expansion based on the cost of each node and estimated values from the heuristic evaluation function. The A* algorithm performs well in finding the shortest path problem but has lower sampling efficiency in high-dimensional spaces and may be limited for complex robotic arm path planning problems. The APF method [8] is prone to getting trapped in local optima during path planning. Parameters such as the weights of goal attraction and obstacle repulsion are difficult to adjust, especially in complex scenarios and tasks where experience or trial-and-error tuning is required. The RRT [9] algorithm is a type of random sampling algorithm that generates nodes through random sampling and constructs paths by connecting nodes and expanding a search tree. The RRT algorithm has good sampling efficiency and global search capability, making it suitable for high-dimensional and complex spatial environments. It gradually expands the search tree through sampling and growth and optimizes and prunes the paths to obtain robotic arm paths that satisfy the constraints.

In terms of planning the initial path of the robotic arm, the rapidly exploring random tree (RRT) algorithm and its variants are capable of searching for efficient paths in high-dimensional spaces and have been widely used in the field of robotic arm path planning [10]. Li Q. [11] proposed an improved informed-RRT* for obstacle avoidance path planning of a six-degree-of-freedom robotic arm but informed-RRT* relies on ellipses for dependency-inspired sampling, which makes it difficult to handle high-dimensional spaces. Meng B. H. [12] proposed a W-space target-biased RRT* for robotic arms, but the exploration is inefficient and prone to local extremes. Qi J. [13] proposes a path-planning algorithm based on the RRT*FN for robotic manipulators. This algorithm improves the real-time requirement of RRT*FN for path planning in robotic arms. Yi J. [14] proposed improved P_RRT* to improve the convergence efficiency of robotic arm path planning. Existing variant RRT* based robotic arm path planning studies still have drawbacks, such as poor goal orientation, generating many useless nodes, and easily falling into local extremes. To solve the above problems, this paper proposes an intelligent beacon sampling method for sampling joint RRT*Smart with improved fusion A*’s cost function. In terms of adapting to dynamic environments, K. Naderi et al. proposed an algorithm called Rt-RRT* [15] that can quickly generate efficient paths in dynamic environments. However, since it only returns partial paths, multiple path re-planning iterations are still required. Adiyatov et al. [16] proposed the RRT*FND algorithm, which utilizes heuristic methods for reconnecting and regrowing to rapidly generate efficient paths in dynamic environments. GA-RRT [17] adds target bias and a new sampling method to the RRT. It achieves motion planning for dual manipulators, but the improved RRT does not generate optimal paths, and the path optimization is not ideal. VT-RRT [18] improves the dynamical step size and target on the basis of RRT, achieving path planning for dual manipulators. However, it is prone to getting stuck in local optima, and the paths generated are not optimal. It simply utilizes RRT to quickly find feasible paths for dual-manipulator cooperation. Spline-RRT* [19] realizes path planning for dual manipulators, but it is not suitable for rapidly generating effective paths in dynamic environments and has long running times. Improved RRT* [20] creates four random trees and adds repulsive potential energy between them in the potential energy function. This method aims to swiftly generate effective paths in dynamic environments, but it is prone to getting stuck in local optima and has less than ideal path optimization for manipulator arms. Shao [21] used cubic spline interpolation on the trajectory points generated by the RRT-Connect path planning algorithm to complete the planning of dual-arm assembly tasks. However, this method only finds feasible paths and not optimal paths, and the effectiveness of the path optimization method is average. Chen [22] utilized the excellent solution ability of the RRT-Connect algorithm in complex environments, but the paths generated were not optimal, and the optimization method used cubic B-splines with average effectiveness.

However, the paths derived based on sampling algorithms such as RRT classes need to be trajectory optimized. Trajectory optimization is the basis for controlling the movement of the robotic arm, and the quality of the trajectory has an important impact on the completion of the operation. Paths based on sampling planning only have spatial information and cannot be used as input for motion control. Trajectory planning transforms paths into expressions about time while satisfying dynamical properties, such as velocity and acceleration range limits and smoothing. In addition, as a prerequisite for trajectory tracking control, trajectory planning affects the accuracy of robot trajectory tracking control [23,24]. Cui et al. [25] utilized fifth- and sixth-order polynomial interpolation to approximate the joint motion trajectory of a free-floating space robot. They employed sequential quadratic programming to optimize and solve the trajectory of joint hinge motion [26]. However, to achieve higher accuracy and smoothness, the main methods used are B-spline curves and non-uniform rational B-spline (NURBS) curves [27]. In previous research, it was found that polynomial interpolation trajectory planning methods can achieve effective obstacle avoidance and rational trajectory selection for robot arms. However, these methods cannot guarantee the attainment of trajectories with optimal performance.

The following Table 1 shows a comparative analysis of path planning in the existing literature.

From Table 1, it can be observed that the path optimization methods of all algorithms are relatively poor, requiring significant improvements. The dynamic path planning method of RRT*FND is relatively advanced, but it still falls short of optimality, thus hindering the achievement of optimal cooperation between dual robotic arms. Sampling methods, such as random sampling or goal bias, as well as the heuristic sampling of improved informed-RRT*, are prone to getting trapped in local optima. By addressing these issues, effective motion planning for dual robotic arm collaboration can be quickly achieved.

The RRT*Smart [28] algorithm has good convergence and an excellent sampling mechanism. However, it has low exploration efficiency, cannot adapt to dynamic environments, and generates non-smooth and constraint-unaware paths for robotic arms. In this paper, we introduce an optimal dynamic motion planning algorithm for robotic arms called RRT*Smart-AD, which is based on the RRT*Smart algorithm. RRT*Smart-AD (rapid-exploring random tree * smart-adaptive regional dynamics) was specifically designed for cooperative dynamic motion planning with dual robotic arms. The improvements in RRT*Smart-AD can be divided into three parts:

(1) In terms of initial path planning, the following enhancements were made to the RRT*Smart algorithm:
  - Dynamic A* evaluation function sampling.
  - Maximum number of fixed nodes by node pruning.
(2) Adaptation to dynamic environments involves:
  - Path detection and judgment in dynamic regions.
  - Path repair.
  - Goal-guided regrowth.
(3) Improvements in trajectory planning include:
  - Trajectory optimization using a combination of PSO and quintic non-uniform rational B-splines (NURBSs).

The sampling mechanism of the RRT*Smart algorithm is suitable for optimizing the original paths but has insufficient exploratory capability. Dynamic A* evaluation function sampling has stronger exploratory capability. A maximum number of fixed nodes can be found by deleting the nodes that do not work for the optimal path in spatial exploration and reducing the nodes of the tree via node pruning, which improves the computational efficiency. Path collision detection in dynamic environments can be increased, and path repair and goal-guided regrowth can quickly generate new valid paths in dynamic environments. With trajectory optimization using a combination of PSO and quintic NURBSs, one can add constraints such as the maximum speed of the robotic arm to make the path smoother and more stable.

Compared with RRT*Smart and RRT*, the solutions obtained using RRT*Smart-AD enable robotic arms to track trajectories better, as the trajectories are straight and contain fewer waypoints. Additionally, the speed of generating efficient paths is faster. By treating the moving robotic arm as a dynamic obstacle to updating the effective path, RRT*Smart-AD is more suitable for the coordination of dual robotic arms. Therefore, it provides a more effective motion planning solution compared with RRT*Smart and RRT*. We demonstrate the effectiveness of our algorithm on a simulated dual robotic arm motion planning problem.

## 2. Robotic Arm Motion Model, Collision Detection Method, and RRT*Smart

### 2.1. Dual Manipulator Arms Experimental Platform

**Figure 1:**
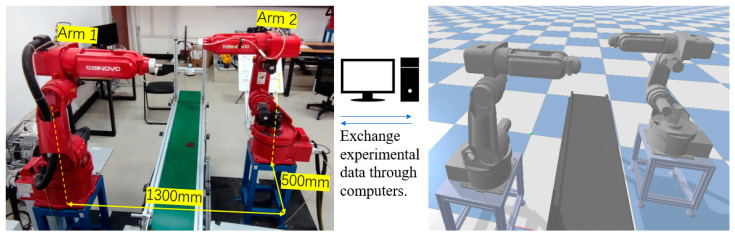
Experimental platform with dual manipulator arms.

As shown in Figure 1, the base coordinate system of primary robotic arm 1 is aligned with the world coordinate system. The base coordinate system of arm 1 in the world coordinate system is [0, 0, 0], and its orientation quaternion is [0, 0, 0, 0]. The base coordinate system of secondary robotic arm 2 is located at [1.3, 0.5, 0] in the world coordinate system, and its orientation quaternion is [0, 0, 1, 0].

**Figure 2:**
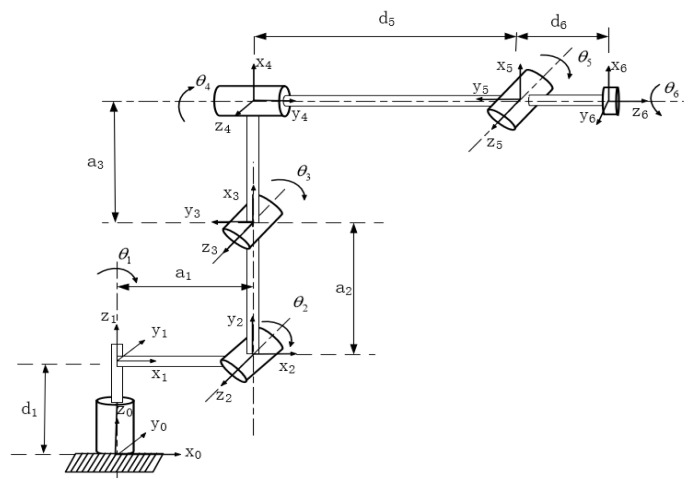
Link coordinate system.

Figure 1 depicts the simulation setup in a Python environment using a pybullet, featuring a conveyor belt as an obstacle. The simulation platform is a 1:1 representation of the real-world setup, and data exchange between the simulation and the actual robotic arm is facilitated through a computer. Table 2 and Figure 2 present the major parameters of the robotic arm’s D-H model.

### 2.2. The Obstacle Detection Methods

Collision detection is a crucial step in path planning within the simulation platform for robotic arms. Traditional approaches often employ bounding box methods [29] to simplify the collision detection process, but this leads to significant errors and lower precision.

To enhance the accuracy of collision detection, we utilized the open-source collision detection library called the Flexible Collision Library (FCL) [30]. The process involves converting the unified robot description format (URDF) model of the robotic arm into an FCL BVH model by triangulating the STL files that represent the arm’s geometry. Joint rotations were accounted for, and collision detection was performed by examining distances between the triangular meshes of the individual joints. The BVH algorithm was employed to reconstruct the robot model.

In the RRT*Smart-AD algorithm, random sampling points are generated in the joint space and transformed into corresponding pose configurations using forward kinematics. By updating the joint poses, collisions between the robot arm and itself, as well as obstacles, can be detected.

**Figure 3:**
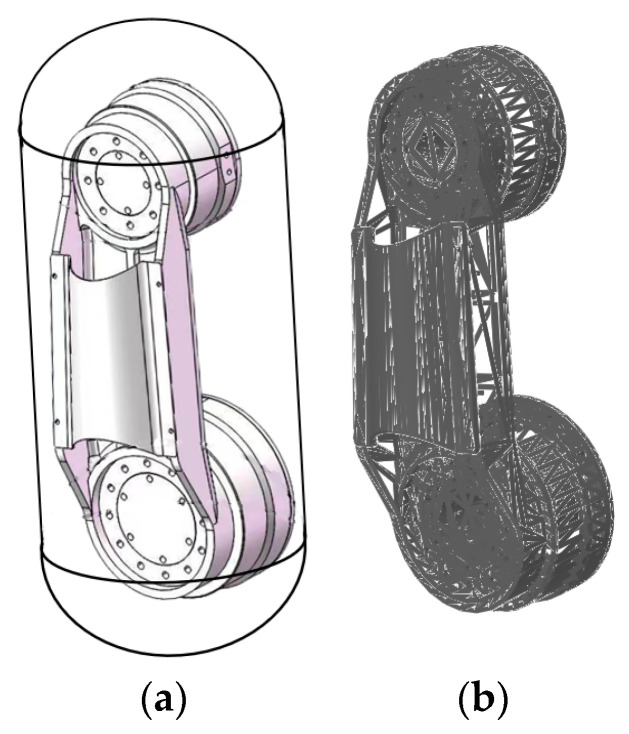
Comparison of collision detection methods for joint 3 components. (**a**) bounding box methods, (**b**) Triangular grid combined with FCL.

Figure 3 depicts a comparison of the collision detection methods for the components of joint 3 in the robotic arm discussed in this paper. As shown in Figure 3a, the bounding box method was utilized, where a cylindrical and a spherical bounding volume were created outside the robotic arm to approximate collisions. However, this method exhibits lower precision and is not suitable for practical engineering applications. In contrast, Figure 3b illustrates the collision detection model established using the method proposed in this paper, which is more aligned with real-world engineering applications.

### 2.3. The RRT*Smart Algorithm

The RRT algorithm is a path-planning algorithm that rapidly explores the solution space and finds feasible paths through random sampling and tree structure expansion. Initially, the algorithm initializes the starting point as the root node and creates an empty tree. Then, in each iteration, the algorithm generates a random point as the target and finds the nearest node to the target in the tree. It extends a certain distance from the nearest node toward the target, creates a new node, and performs collision detection. If the new node does not collide with obstacles, it is added to the tree and connected to the nearest node. If a new node reaches the target, a path connecting the starting point and the target is found. In this way, the RRT algorithm efficiently explores the solution space and finds feasible paths.

RRT* is an improved version of the RRT algorithm that builds upon the original RRT to further enhance the path quality and convergence. RRT* improves the path quality by utilizing an optimal distance metric and selecting optimal connections. It generates excellent path solutions by optimizing and pruning nodes in the tree. By combining node optimization and pruning, RRT* can generate paths that are closer to the optimal solution. In comparison with the traditional RRT, RRT* allows for node rewiring to optimize the path. This means that RRT* can select shorter paths between nodes that do not simply extend from a single node, but rather extend from the optimal point by searching among all the nodes. RRT* offers improvements over RRT in terms of the path quality, convergence, and ability to approximate optimal paths. By optimizing and pruning nodes, employing rewiring strategies, and considering nodes from the entire tree, RRT* can generate paths that are closer to the optimal solution.

RRT*Smart is an extension of RRT* that operates similarly to RRT* but with the additional feature of removing redundant nodes from the initial path once it is found. RRT*Smart has demonstrated better path convergence compared with RRT*. Furthermore, it identifies intelligent beacon samples for path improvement. This sampling differs from random sampling, as it is biased toward optimizing the beacon nodes along the path. It utilizes nearby obstacle beacon nodes to set the distance for intelligent exploration around the selected beacon. Once RRT*Smart finds a shorter path, it performs the path optimization process again to generate new beacon nodes. The main functions for describing the representation of robotic arm path planning are as follows:

Sample: If an initial valid path is not found, this function generates random joint angle positions $Z_{\mathrm{rand}}$ from the available joint space $Z_{\mathrm{free}}$ in the robotic arm’s collision-free joint space. Otherwise, it searches for a random node within a distance of R around the obstacle beacon nodes $Z_{\mathrm{beacons}}$ near the valid path.

Nearest: This function returns the node $Z_{\mathrm{nearest}}$ in the tree T = (V, E) that is closest to the node $Z_{\mathrm{rand}}$ based on the cost function.

Steer: This function takes inputs $Z_{\mathrm{rand}}$ and $Z_{\mathrm{nearest}}$ grows Δq along the direction from $Z_{\mathrm{rand}}\to Z_{\mathrm{nearest}}$ to obtain $Z_{\mathrm{new}}$, where Δq is the step length distance.

CollisionDetect: This function is used to detect collisions in the branches of the tree, and it returns true if the path $Z:\left[0,\;T\right]$ for all t = 0 to t = T is within the obstacle-free region $Z_{\mathrm{free}}$.

Near: This function returns all the nodes in the tree that satisfy the condition of being within a defined neighborhood distance β of $Z_{\mathrm{new}}$.

InsertNode: This function adds the node $Z_{\mathrm{new}}$ to the tree $T=\left(V,\;E\right)$ and connects it to the node $Z_{\min}$ as its parent node.

PathOptimization: Input $\left(T{,\;Z}_{\mathrm{init}}{,\;Z}_{\mathrm{goal}}\right)$, where this function determines an optimized path by directly connecting nodes that are mutually visible along the path and updates the set of beacon nodes $Z_{\mathrm{beacons}}$.

ExtractPath: This function extracts the optimal path from the collection of trees. 

A pseudo-code describing RRT*Smart is shown in Algorithm 1.

**Algorithm 1** $T=(V,E)\;\leftarrow RRT*Smart{(Z}_{\mathrm{init}})$

$\begin{array}{l} 1\;T\leftarrow \mathrm{InitializeTree}\left(\right)\\ 2\;T\leftarrow \mathrm{InsertNode}(\phi ,Z_{\mathrm{init}},T)\\ 3\;for\;i\;\mathrm{in}\;\mathrm{range}\left(N\right):\\ 4\;\;if\;\left(i\;-\;n\right)\;\%\;b\;=\;0:\\ 5\;\;Z_{\mathrm{rand}}\;\leftarrow {\mathrm{Sample}(i,Z}_{\mathrm{beacons}})\\ 6\;\;else\\ 7\;\;Z_{\mathrm{rand}}\leftarrow \mathrm{Sample}\left(i\right)\\ 8\;\;Z_{\mathrm{nearest}}\;\leftarrow \;\mathrm{Nearest}(T{,\;Z}_{\mathrm{rand}}):\\ 9\;\;\left(X_{\mathrm{new}}{,\;U}_{\mathrm{new}},\;T_{\mathrm{new}}\right)\;\leftarrow \;\mathrm{Steer}\;\left(Z_{\mathrm{nearest}}{,\;Z}_{\mathrm{ranad}}\right)\;\\ 10\;\;if\mathrm{ObstaclefreeX}\left(Z_{\mathrm{new}}\right)\;:\\ 11\;\;\;Z_{\mathrm{near}}\leftarrow \mathrm{Near}(T,Z_{\mathrm{new}},\left|V\right|)\\ 12\;\;\;Z_{\min}\leftarrow \mathrm{Chooseparent}\;(Z_{\mathrm{near}},Z_{\mathrm{nearest}},Z_{\mathrm{new}},X_{\mathrm{new}})\\ 13\;\;\;T\leftarrow \mathrm{InsertNode}(Z_{\min},Z_{\mathrm{new}},T)\\ 14\;\;\;T\leftarrow \mathrm{Rewire}\left(T,Z_{\mathrm{new}},Z_{\mathrm{near}}\right)\\ 15\;\;\;if\;\mathrm{InitialPathFound}:\\ 16\;\;\;\;\;\;n=i\\ 17\;\;\;\left(T,\mathrm{directcost}\right)\leftarrow \mathrm{PathOptimization}\left(T,Z_{\mathrm{init}}{,\;Z}_{\mathrm{goal}}\right)\\ 18\;\;\;if{\;\mathrm{directcost}}_{\mathrm{new}}{<\;\mathrm{directcost}}_{\mathrm{old}}\\ 19\;\;\;\;\;{\;Z}_{\mathrm{beacons}}\leftarrow \mathrm{PathOptimization}(T,Z_{\mathrm{init}},Z_{\mathrm{goal}});\\ 20\;\mathrm{return}\;\;\mathrm{ExtractPath}(T,Z_{\mathrm{init}},Z_{\mathrm{goal}}) \end{array}$



## 3. RRT*Smart-AD Algorithm

The RRT*Smart-AD algorithm can be divided into three parts. First, it involves initial cooperative obstacle avoidance path planning. Second, it detects whether the path in the dynamic environment becomes invalid and re-plans an effective path. Third, it utilizes quintic NURBS trajectory optimization. In the first part, the RRT*Smart algorithm is enhanced by incorporating dynamic A* function sampling and pruning the path based on a maximum node limit. This method improves the efficiency of finding the initial path and reduces the algorithm’s runtime. The second and third parts determine the validity of the path in the dynamic region. They generate new effective paths through reconnecting and regrowing, enhancing adaptability to dynamic environments. Lastly, the RRT*Smart-AD utilizes PSO to optimize the discrete point path generated by the RRT*Smart-AD algorithm, resulting in a motion trajectory for the robotic arm.

### 3.1. Improvements in Sampling Methods

#### 3.1.1. Dynamic A* Evaluation Function Sampling

For most variations in the RRT* algorithm, it is not suitable to sample the goal point with a certain probability. This is because after generating the initial path (with the goal point already added to the tree set), the closest point in the tree to the sampled point will always be the goal point itself (with a distance of 0). The A* algorithm’s heuristic function can enhance the convergence efficiency of the path. To address this issue and improve sampling thoroughness, the RRT*Smart-AD algorithm combines a dynamic A* evaluation function sampling method.

The algorithm generates a sequence of random sampling points and sorts all nodes based on the cost function from smallest to largest. The optimal point in this sequence becomes the starting point for exploration. If collisions prevent expansion, a suboptimal point is selected if the optimal point fails to expand. To prevent falling into local optima, node sparsity [31] is employed to avoid repeatedly searching the same regions, even if they have optimal values. By improving the sampling method, effective nodes close to the shortest distance between start and end points can be selected, leading to convergence toward the target. Removing duplicate values and nodes with small distances prevents getting trapped in local search, increasing algorithm stability.

(1) f(n)=k⋅g(n)+h(n)

(2) $$k=\frac{\left(-1+e^{-2u}\right)w_{\max}}{1+e^{-2u}}+\frac{w_{\max}}{2}+w_{\min}+b\cdot t\cdot \sin\left(b\cdot t\right)/i$$

(3) $$u=a\cdot \left(t-c\cdot i\right)$$

In these equations, f(n) represents the cost value of the node evaluation, where a smaller value indicates a better evaluation. g(n) represents the Euclidean distance from the start point $Z_{\mathrm{init}}$ to the current node $Z_{\mathrm{new}}$, while h(n) represents the Euclidean distance from the current node $Z_{\mathrm{new}}$ to the goal point $Z_{\mathrm{goal}}$. k is the dynamic forward coefficient, where a larger value indicates a stronger bias toward the goal point. The value of k can be determined using Formulas (2) and (3). a denotes the value of slow descent in finding the initial path and is in the range [0.001, 0.1]; in this study, we used 0.08. b is the weight to keep the data scale consistent and is in the range [0.01, 0.09]; in this study, we used 0.01.

**Figure 4:**
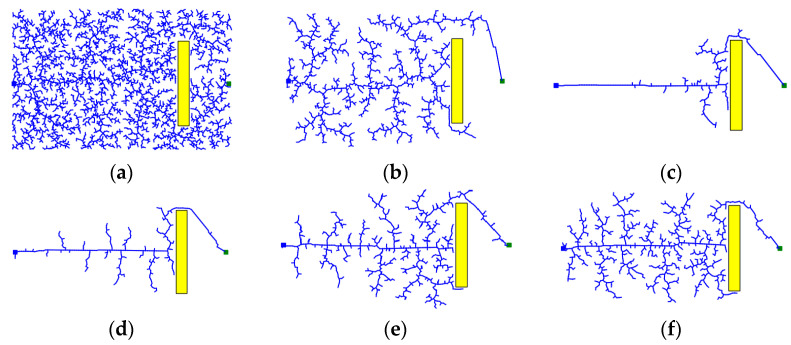
Comparison of sampling methods. (**a**) RRT random sampling; (**b**) Target bias sampling; (**c**) A* sampling (k = 3); (**d**) A* sampling (k = 1.5); (**e**) A* sampling (k = 1.2); (**f**) A* sampling (k = 1).

The path search process of the RRT*Smart-AD algorithm can be divided into global path search and local optimization search. Before finding a valid path, it is necessary to find a relatively optimal valid path. In the fusion of the A* evaluation function sampling method, stronger guidance is required, which means a larger value of the dynamic forward coefficient k, as shown in the Figure 4f plot. When an effective path is found, it is important to explore the shortest path thoroughly and increase the randomness of exploration. In this case, a smaller value of the k coefficient is needed, as shown in the Figure 4c plot.

To better demonstrate the efficiency of the sampling methods proposed in this paper, we conducted experimental comparisons of three sampling methods in a two-dimensional setting, as shown in Figure 4. Among these three methods, the random sampling method represents the standard RRT sampling method, where nodes are randomly generated in the space, resulting in lower efficiency. The goal-bias method sets the target point as a sampling node with a certain probability, which provides strong guidance but is susceptible to local optima, and still employs random sampling when obstacles are present. The dynamic A* evaluation function, which is based on the goal-bias method, selects more optimal sampling points, enhancing the guidance. The addition of the node sparsity method prevents sampling from getting stuck in local optima. In the environment depicted in Figure 4, we conducted experiments starting from the initial point using the three different sampling methods and stopped when reaching the target point on the right. We compared the number of sampled nodes reached and the exploration efficiency. The blue and green squares represent the starting and target points, respectively, while the yellow rectangles represent obstacles. The experimental results are shown in Figure 4, where the blue lines represent the sampling paths.

From Figure 4, it can be observed that the A* evaluation function sampling method had the fewest number of nodes and a higher exploration efficiency. The standard RRT* sampling adopts random sampling, while the goal-bias RRT algorithm loses its guidance when obstructed by obstacles and still employs random sampling, similar to the standard RRT* algorithm. The proposed improved A* evaluation function sampling method considers both the sufficiency of sampling exploration and goal guidance. The larger value of the dynamic forward coefficient ensures a more thorough exploration along the shortest path. Increasing the coefficient strengthens the guidance without losing its effectiveness due to obstacles. The generated iterative points after using the improved A* evaluation function sampling method converge faster, thus improving the efficiency of the path optimization in the iterative space.

#### 3.1.2. Maximum Number of Fixed Nodes via Node Pruning

**Figure 5:**
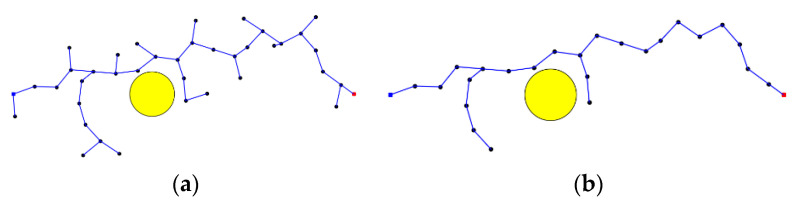
Before and after tree downsizing. (**a**) Before downsizing; (**b**) After downsizing.

The RRT*FN algorithm [32] achieves node deletion by randomly removing leaf nodes when the number of nodes reaches the predefined maximum value. This method requires relatively less memory during the path search process, reducing the computation time. However, it has lower convergence accuracy, and the random node deletion after reaching the maximum number of nodes increases the computation time. To achieve higher convergence performance, this proposed algorithm further improves the node deletion method. Specifically, all the child nodes in the tree, except for the end sub-nodes on the effective path, are deleted. Figure 5 illustrates the principle, The blue dot on the left is the start point, the red dot on the right is the end point, the black dot is the sampling point, and the yellow circle is the obstacle. The blue line segment is the sampling path, where (a) represents the nodes in the tree before any deletion, and (b) shows the tree after deleting the child nodes. By shrinking the maximum number of fixed nodes in the sampling tree, the nodes in the tree that do not contribute to the optimal path can be reduced, thus improving the operational efficiency of the algorithm.

### 3.2. Dynamic Motion Planning Strategy

This subsection describes methods for determining the detection of path invalidation and for quickly generating a new valid path after the original path has failed.

#### 3.2.1. Path Detection in Dynamic Regions

**Figure 6:**
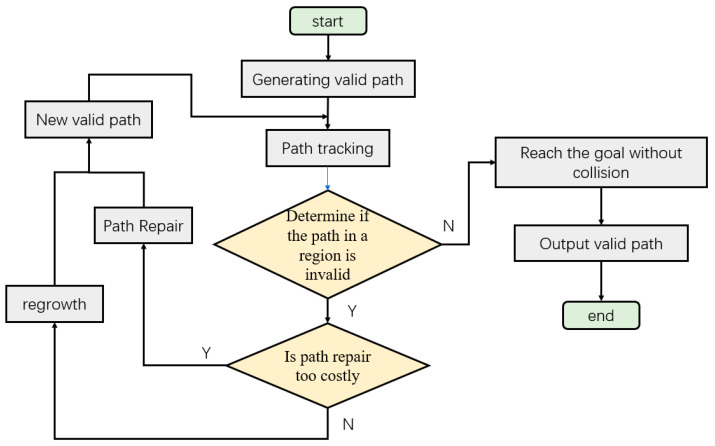
The flowchart of the Dynamic motion planning strategy.

In dual-arm systems, dynamic path-planning methods can coordinate the motion of each robot arm to ensure their collaboration and cooperative work. Among the existing methods for detecting path failure in dynamic path planning algorithms, the RRT*FND algorithm detects whether the entire path is valid and generates new paths in real time. However, real-time collision detection along the entire path can greatly impact the efficiency of reaching the target point, as failure in the latter part of the path does not necessarily affect tracking the unaffected path in the front. Additionally, in scenarios with long paths and numerous obstacles, regrowth is required for almost every tracking step of the path. To address this, a dynamic region path recovery and regrowth method is proposed. It determines whether path recovery and regrowth are necessary within the tracked point’s region. The initially planned path primarily serves as a guiding path and a means to determine whether a valid path can reach the target point. The flowchart for dynamic region path recovery and regrowth within the region is shown in Figure 6.

Assume the optimized motion path of the robotic arm for a single joint is $X=\left[x_{1},x_{2},\ldots ,x_{p}\right]\;$. R represents the initial path angle distance. $r_{i}$ is the weight of R, which ensures the length of the tracked path and ranges between [0.2, 0.4], with a value of 0.3 in this case. For a given point during the motion, if the number of predicted positions within a distance $r_{i}R$ is n, then for that point, there exists a set $U=\left[x_{i},x_{i+1},\ldots ,x_{i+n}\right]$. The collision detection method described in Section 2.2 is used to determine whether the paths within the region defined by $U=\left[x_{i},x_{i+1},\ldots ,x_{i+n}\right]$ are valid or not.

**Figure 7:**
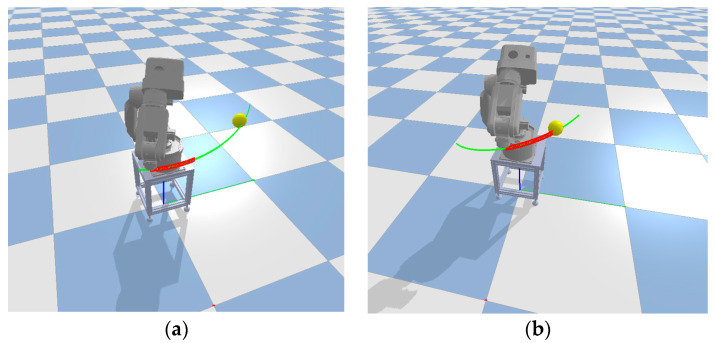
Dynamic area range to determine whether the path is invalid. (**a**) no collision at dynamic detection distance, (**b**) collision at dynamic detection distance.

The idea of determining path failure in a dynamic region is to avoid tracking the entire path and instead focus on a portion of the path defined by the initial joint path distance. The joint path distance refers to the sum of the angle changes of the robot arm’s joints from the starting point to the endpoint. This method sacrifices the predictive distance to reduce the computational complexity and improve the path execution efficiency. Experimental results showed that this method has better performance in dynamic path planning. As shown in Figure 7, the green path represents the initial path, while the red path indicates the dynamic region-based path failure detection. The yellow sphere is a moving obstacle. In Figure 7a, no path failure is detected, and the path can be continued to be tracked. However, in Figure 7b, path failure is detected, requiring path repair or regrowth to generate a new feasible path at that point.

#### 3.2.2. Path Repair

In Section 3.2.1, when the collision detection method detects a failure in the path, a new valid path needs to be generated. When the path collides with an obstacle and the failure is relatively simple, a simple and effective method is proposed to handle the failure of short-distance, simple paths.

**Figure 8:**
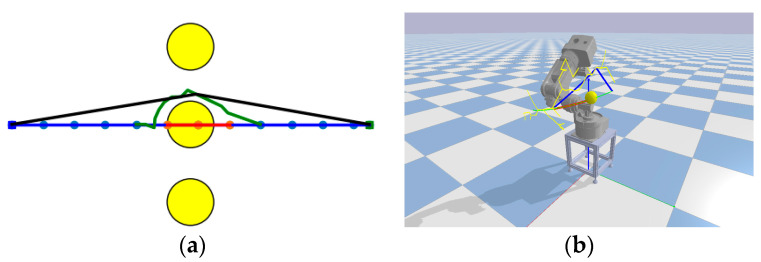
Path repair principle and robotic arm simulation path repair. (**a**) Demonstration of the principle of path repair. (**b**) Demonstration of the principle of path repair 6-dof robotic arm.

First, the failure point node of the path is identified to determine whether it meets the conditions for path repair: the number of path repairs and the distance to be repaired are calculated. The path repair condition is met when $N_{r}\leq P_{r}$ and $D_{r}$ < $r_{i}R$, where $P_{r}$ represents the maximum number of repairs allowed (ranging from 2 to 5, with a value of 2 in this study). To ensure that the repair distance is not too large, $r_{i}$ was set to 0.1. Path repair significantly reduces the time required to obtain a new valid path. The principle is illustrated in Figure 8a, where a single breakpoint path repair is performed in a two-dimensional space.

Step 1: The blue path starts from the blue square on the left and ends at the green square on the right. When a path disruption occurs within the tracking point area due to an obstacle (yellow circle), the disrupted path is shown in red. The invalid nodes that intersect with the obstacle are disconnected from the other valid nodes, and these invalid nodes are discarded.

Step 2: Two failure points in the invalid path are selected, the distance between them is calculated, and a path repair is performed, resulting in the green path segment.

Step 3: The process continues by searching for path breakpoints and repeating step 2 until the path is repaired. This repair process may introduce redundant nodes. To eliminate these redundant nodes, a greedy strategy is applied, resulting in the black path. The greedy strategy is applied from the start to the endpoint.

Figure 8b shows a simulation of path repair for a robotic arm. The green path represents the initial path, the red path indicates the dynamic region-based path failure, the yellow path represents the sampled points, and the blue path represents the repaired path.

#### 3.2.3. Regrowth

In the RRT variation algorithm, Extend-RRT [26] proposes a method for addressing path failures by removing the failed nodes in the original search tree and regrowing the path. Rt-RRT* introduces an online tree rewiring strategy that allows the tree root to move with the agent without discarding previously sampled paths. RRT*FND extends this method by assigning node numbers for invoking the unaffected nodes to generate new valid paths. Building upon this, a regrowth method is proposed that incorporates the original path guidance.

**Figure 9:**
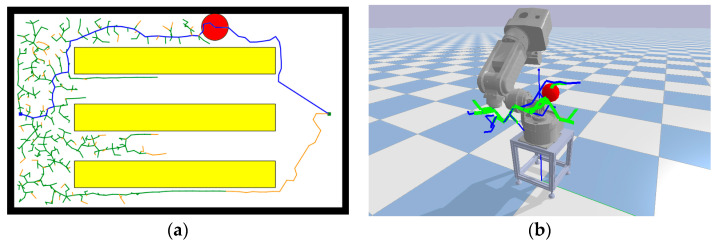
Path regrowth 2D simulation and path regrowth of robotic arm. (**a**) Demonstration of the principle of path Regrowth. (**b**) Demonstration of the principle of path Regrowth 6-dof robotic arm.

When there are frequent path repair attempts and the repair distance is long, the probability of obtaining an optimal or suboptimal path using path repair methods becomes small. In such cases, the valid nodes in the original tree are retained, and regrowth is performed on the unaffected nodes. The invalid nodes that intersect with obstacles in the disrupted path are disconnected from the other valid nodes and discarded. The failed path still serves as a guide by incorporating the unaffected points from the initially failed path into the target bias for path guidance. The regrowth principle is demonstrated in a two-dimensional visual representation in Figure 9a. When the initial path (blue path) encounters a new obstacle (red circle), and thus, fails, the failed nodes are removed, and the original tree is explored in space until a valid path is found. The green path represents the unaffected nodes, while the orange path represents the regrown nodes.

In Figure 9b, the regrowth process is demonstrated in a simulated space for a robotic arm. The green path represents the original tree collection. After the appearance of a red spherical obstacle, the original tree collection and path become invalid. Utilizing the path regrowth method with guidance effect, a new valid tree collection is generated based on the original failed tree, as shown in blue.

**Figure 10:**
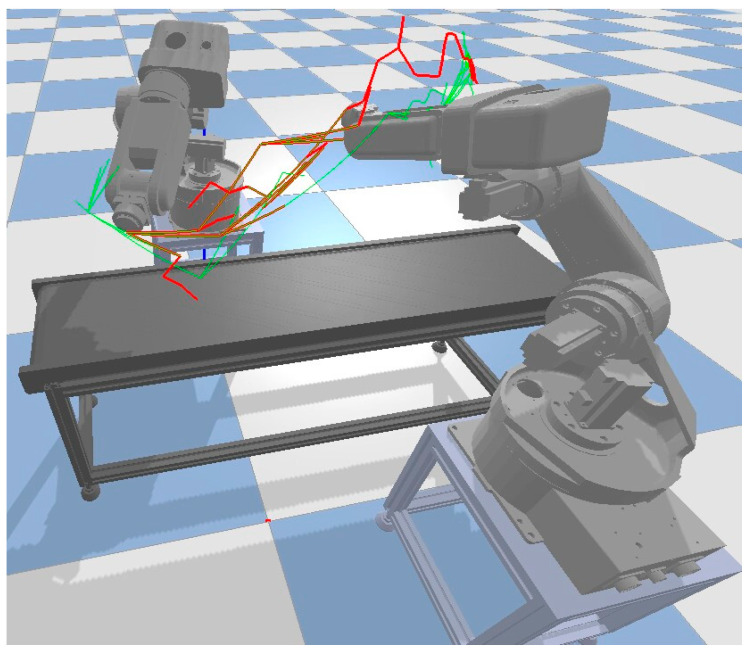
Re-growth with double robotic arm cooperation.

When the dual robotic arms collaborate and a collision is predicted after the robot arms have moved to a trajectory point, it is necessary to disconnect the tracked points and their connected child nodes, as well as the invalid nodes that collide with obstacles (such as the moving robotic arm 2). As shown in Figure 10, the green paths represent the initial path sampling points, the gold points represent the preserved sampling points from the old tree after collision prediction, and the red points represent the new sampling points generated during regrowth. The regrowth method avoids duplicating nodes and fully utilizes the information from the old tree.

### 3.3. PSO Combined with Quintic-NURBS-Optimized Trajectories

To ensure that the generated path meets the practical requirements of a robotic arm, a combined approach of the PSO algorithm and quintic NURBS optimization for robotic arm trajectory is proposed. The discrete point path obtained from the RRT*SMART-AD algorithm is smoothed. The method combines quintic NURBS curves and the particle swarm optimization (PSO) algorithm to generate functions for the parameters of the joints, considering the constraints of the robotic arm. This approach aims to obtain a high-order continuous motion trajectory. Additionally, while optimizing the path for smoothness, the algorithm also minimizes the operational distance of the robotic arm.

In robotic arm path planning, the quintic B-spline curve has higher-order continuity, i.e., the curvature changes between the nodes on the curve are continuously smooth. The smoothness and continuity of the robotic arm during its motion are very important. Robotic arm path planning usually needs to consider the joint constraints and motion limitations of the robotic arm, and the use of the quintic B-spline curve with higher degrees of freedom can better meet these constraints and limitations. The shape of the quintic B-spline curve can be flexibly controlled by adjusting the positions and weights of the control points. In the robotic arm path planning, the parameters of the control points can be adjusted to achieve the fine-tuning and optimization of the path so that the robotic arm can reach the target position more accurately and smoothly without collision during the movement.

#### 3.3.1. The Quintic NURBS Principle

In the path planning of a robotic arm, B-spline curves exhibit high-order continuity, meaning that the curvature changes between each node on the curve are continuously smooth. The smoothness and continuity of the robotic arm’s motion are crucial [33]. Furthermore, path planning for the robotic arm typically needs to consider the joint constraints and motion limitations of the arm. Using quintic B-spline curves with higher degrees of freedom can better satisfy these constraints and limitations. The shape of the fifth-degree B-spline curve can be flexibly controlled by adjusting the positions and weights of the control points [34]. This enables the robotic arm to move smoothly and precisely without collisions, reaching the desired positions.

The basis functions in NURBSs [35] are described as Equations (4) and (5):

(4) $$C\left(\xi \right)=\frac{1}{\sum {}_{i=0}^{n}N_{i,p}\left(\xi \right)w_{i}}\sum_{i=0}^{n}N_{i,p}\left(\xi \right)w_{i}P_{i}$$

(5) $$\begin{array}{l} N_{i,0}\left(\xi \right)=\left\{\begin{array}{c} 1\;\mathrm{if}\;\xi_{i}\leq \xi \leq \xi_{i+1}\\ 0\;\;\;\;\;\mathrm{otherwise} \end{array}\right.\\ N_{i,p}\left(\xi \right)=\frac{\xi -\xi_{i}}{\xi_{i+p}-\xi_{i}}N_{i,p-1}\left(\xi \right)+\frac{\xi_{i+p}-\xi_{i}}{\xi_{i+p+1}-\xi_{i+1}}N_{i+1,p-1}\left(\xi \right) \end{array}$$

In these equations, p represents the dimension of the spline. ξ denotes the node value. $N_{i,p-1}\left(\xi \right)$ represents the i-th basis function. $P_{i}$ corresponds to the control points, while $w_{i}$ denotes the weight values. The weight values determine the degree of control that the control points have on the curve.

#### 3.3.2. Particle Swarm Optimization Algorithm

**Figure 11:**
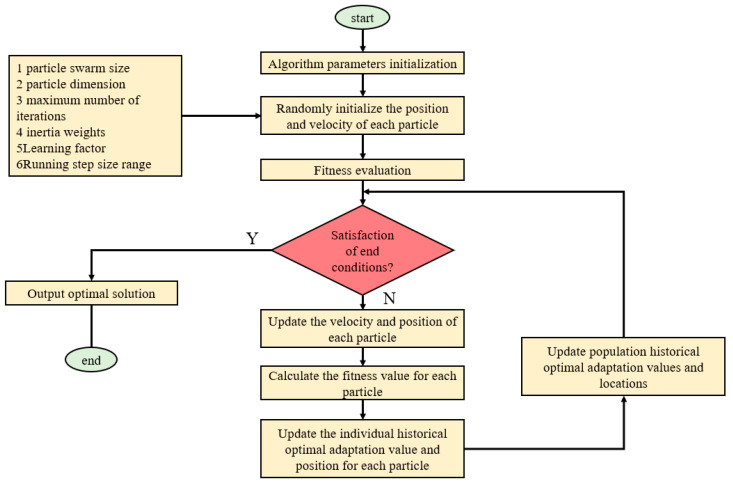
The flowchart of the PSO algorithm.

The PSO algorithm is a multi-parameter optimization technique based on swarm intelligence. It draws inspiration from artificial life and evolutionary computation theories. The PSO algorithm simulates the migration and learning process of particles in the search space to find the optimal combination of parameters. During the iteration process of PSO, the particle swarm gradually moves closer to the target area, searching for the optimal solution through information exchange and collaborative behavior. Each particle optimizes its search by tracking the best solution it has found so far and the best solution found by the entire swarm. The PSO algorithm is simple to implement and does not rely on the gradient information of the objective function, making it suitable for various types of optimization problems. Figure 11 shows the flowchart of the PSO algorithm:

#### 3.3.3. PSO Combined with Quintic NURBSs to Optimize the Robotic Arm Trajectory

The optimization approach is as follows:

(1) Introducing constraint conditions: In practical applications, a robotic arm needs to satisfy a series of constraint conditions, such as collision avoidance, maximum joint acceleration limits, and maximum velocity limits. By incorporating these constraint conditions into the optimization of quintic NURBSs, the resulting trajectory can better adhere to real-world application requirements.

(2) Number of control points: In quintic NURBSs, the more control points used, the more accurately the curve can match the target shape. However, this can also lead to overfitting and increased complexity of the curve. Therefore, a balance needs to be struck in selecting the number of control points.

(3) Adjustment of weight values: In quintic NURBSs, each control point contributes to the shape of the curve, and the extent of this influence is determined by the weight value assigned to that control point. Optimizing the weight values ensures a more reasonable and balanced contribution of control points toward the desired trajectory.

To determine the appropriate number of control points and weight values that satisfy the external constraints of the robotic arm and obtain an optimal robotic arm trajectory with a minimized distance, the PSO is employed as a multi-objective parameter optimization algorithm. It utilizes the PSO in conjunction with the RRT*Smart-AD algorithm to generate efficient paths (discrete points) and search for the optimal solutions of the two sets of values for the quintic NURBSs, thereby obtaining a distance-minimized path that satisfies the motion constraints of the robotic arm.

Due to the limited constraints of maximum velocity and acceleration in the robotic arm used in this study, only the first-derivative velocity curve and second-derivative acceleration curve of the quintic NURBSs were obtained via differentiation. The set of first-derivative velocity values is denoted as $C^{1}\left(\xi \right)$, and the set of second-derivative acceleration values is denoted as $C^{2}\left(\xi \right)$. The maximum acceleration limit for each joint is denoted as $a_{m}$, and the maximum velocity limit is denoted as $v_{m}$. The joint distance of the path $C\left(\xi \right)$ is obtained to form $S\left(C\left(\xi \right)\right)$. The Q value is constructed, and when the points in $C\left(\xi \right)$ fail the collision detection or when the maximum values of the velocity set $C^{1}\left(\xi \right)$ and acceleration set $C^{2}\left(\xi \right)$ exceed the limits of the robotic arm itself, Q is assigned a very high value. The fitness function of the PSO is given as Equation (6):

(6) $$\begin{array}{l} F\left(x\right)=S\left(C\left(\xi \right)\right)+Q\\ Q=\left\{\begin{array}{c} \mathrm{sys}.\mathrm{maxsize},\;if\;\max\left(C^{1}\left(\xi \right)\right)>v_{m}\;or\;\max\left(C^{2}\left(\xi \right)\right)>a_{m}\;or\;\mathrm{Path}\_\mathrm{invalid}\\ 0,\;\mathrm{otherwise} \end{array}\right. \end{array}$$

The maximum value sys.maxsize mentioned in the equation is the predefined maximum value in Python.

The quintic NURBSs curve is employed as a representation of the motion trajectory. It generates high-order continuous curves that comply with the constraints of the robotic arm, making it suitable for arm planning. The PSO algorithm is utilized to optimize the parameter search process, with the aim of finding the optimal combination of parameters and achieving desirable motion effects. By combining these two methods, an effective and continuous fifth-order motion trajectory is obtained, enabling smoother and more stable arm movements.

### 3.4. Flowchart of the RRT*Smart-AD Algorithm

**Figure 12:**
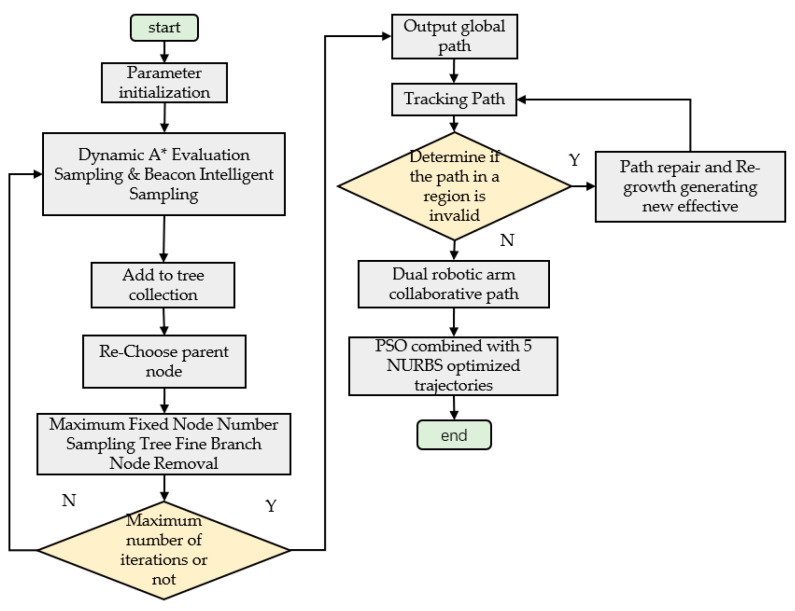
RRT*Smart-AD algorithm flow chart.

Figure 12 shows the flowchart of the RRT*Smart-AD algorithm:

The process of RRT*Smart-AD is outlined in Algorithm 2.

**Algorithm 2** $T=(V,E)\;\leftarrow RRT*Smart-AD{(Z}_{\mathrm{init}})$

$\begin{array}{l} 1\;T\leftarrow \mathrm{InitializeTree}\left(\right)\\ 2\;T\leftarrow \mathrm{InsertNode}(\phi ,Z_{\mathrm{init}},T)\\ 3\;for\;i\;\mathrm{in}\;\mathrm{range}\left(N\right):\\ 4\;\;if\;\left(i\;-\;n\right)\;\%\;b\;=\;0:\\ 5\;\;\;{\;Z}_{\mathrm{rand}}\;\leftarrow {\mathrm{Sample}(i,Z}_{\mathrm{beacons}})\\ 6\;\;else\\ 7\;\;\;{\;Z}_{\mathrm{rand}}\leftarrow A*\_\mathrm{Sampling}\_\mathrm{function}\left(m,i\right)\\ 8\;\;Z_{\mathrm{nearest}}\;\leftarrow \;\mathrm{Nearest}(T{,\;Z}_{\mathrm{rand}}):\\ 9\;\;\left(X_{\mathrm{new}}{,\;U}_{\mathrm{new}},\;T_{\mathrm{new}}\right)\;\leftarrow \;\mathrm{Steer}\;\left(Z_{\mathrm{nearest}}{,\;Z}_{\mathrm{ranad}}\right)\;\\ 10\;\;if\mathrm{ObstaclefreeX}\left(Z_{\mathrm{new}}\right)\;:\\ 11\;\;\;Z_{\mathrm{near}}\leftarrow \mathrm{Near}(T,Z_{\mathrm{new}},\left|V\right|)\\ 12\;\;\;Z_{\mathrm{nin}}\leftarrow \mathrm{Chooseparent}\;(Z_{\mathrm{near}},Z_{\mathrm{nearest}},Z_{\mathrm{new}},X_{\mathrm{new}})\\ 13\;\;\;T\leftarrow \mathrm{InsertNode}(Z_{\min},Z_{\mathrm{new}},T)\\ 14\;\;\;T\leftarrow \mathrm{Rewire}\left(T,Z_{\mathrm{near}},Z_{\mathrm{new}}\right)\\ 15\;\;\;if\;\mathrm{InitialPathFound}:\\ 16\;\;\;\;\;n\;=\;i\\ 17\;\;\;\left(T,\mathrm{directcost}\right)\leftarrow \mathrm{PathOptimization}\left(T,Z_{\mathrm{init}}{,\;Z}_{\mathrm{goal}}\right)\\ 18\;\;\;if{\;\mathrm{directcost}}_{\mathrm{new}}{<\;\mathrm{directcost}}_{\mathrm{old}}\\ 19\;\;\;\;{\;Z}_{\mathrm{beacons}}\leftarrow \mathrm{PathOptimization}(T,Z_{\mathrm{init}},Z_{\mathrm{goal}});\\ 20\;\;\;if\;\mathrm{len}\left(T\right)<\;\mathrm{FN}\;:\\ 21\;\;\;\;T\leftarrow \mathrm{TreeFineBranchNodeRemoval}\left(T\right)\\ 22\;\mathrm{path}\leftarrow \mathrm{ExtractPath}(T,Z_{\mathrm{init}},Z_{\mathrm{goal}})\\ 23\;\mathrm{InitializeRobotic}\_\mathrm{arm}\left(\mathrm{arm}1,\mathrm{arm}2\right)\\ 24\;\mathrm{while}\;\mathrm{Path}\_\mathrm{detection}\;(\mathrm{path}):\\ 25\;\;if\;N_{r}\leq P_{r}\;\mathrm{and}\;D_{r}\leq r_{i}R:\\ 26\;\;\;\mathrm{path}\leftarrow \mathrm{Path}\_\mathrm{repair}(T,Z_{\mathrm{init}},Z_{\mathrm{goal}})\\ 27\;\;else:\\ 28\;\;\;\mathrm{path}\leftarrow \mathrm{Path}\_\mathrm{regrowth}(T,Z_{\mathrm{init}},Z_{\mathrm{goal}})\\ 29\;\mathrm{path}\leftarrow \mathrm{PSO}\_\mathrm{NURBS}(5,\mathrm{Path}) \end{array}$



## 4. Experimental Validation and Analysis of the RRT*Smart-AD Algorithm

Path planning algorithms for robotic arms involve sampling in a six-dimensional space of one-dimensional joints. In contrast, two-dimensional simulations involve sampling in two one-dimensional spaces, offering more intuitive results. To fully demonstrate the superiority of the RRT*Smart-AD algorithm, we designed static and dynamic two-dimensional simulations for the robotic arm. These experiments were compared against other excellent improved RRT*-based algorithms.

To facilitate analysis and ensure practicality, we controlled other unrelated variables to remain consistent while focusing on the algorithm itself. To mitigate the impact of randomness in the RRT sampling algorithms, we performed 50 experiments and compared and analyzed the results.

The computer setup consisted of a 64-bit Windows 10 Professional operating system, an AMD Ryzen 5 2600X six-core processor, a Radeon RX580 graphics card, and a Python programming environment.

### 4.1. Algorithm Performance Comparison

**Figure 13:**
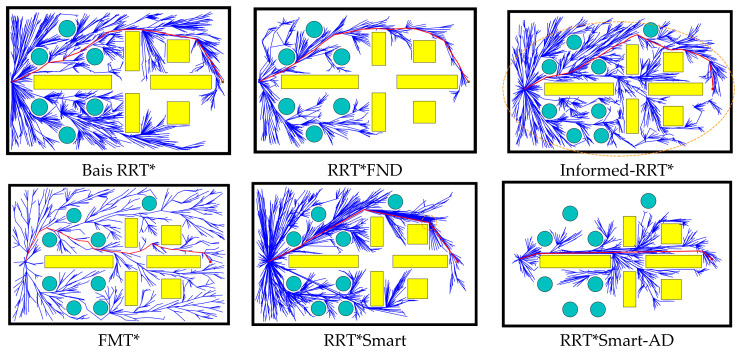
Comparison of static two-dimensional space algorithm path planning.

Setting a complex two-dimensional map (800 × 520) for validating the RRT*Smart-AD algorithm: To verify the performance of the RRT*Smart-AD algorithm and other algorithms in a static obstacle environment, we constructed a complex two-dimensional map with dimensions of 800 × 520. The map is located in the first quadrant, with the start point at [20, 20] and the goal point at [780, 500]. All algorithms shared the same parameters, with a step size of 8 and a goal bias of 0.2. The path planning results of the Bais RRT*, RRT*FND, RRT*Smart, informed-RRT*, FMT*, and RRT*Smart-AD algorithms are shown in Figure 13. The sampled point paths are represented in blue, circles and rectangles are obstacles, and the final solution paths are marked in red, the orange ellipse circle is the dynamic sampling area of Informed-RRT*. For a visual comparison, the average path lengths and runtimes from 50 experiments are listed in Table 3.

From the comparison in Figure 13, it can be observed that for all RRT*-based algorithms without goal bias, when there were many obstacles in the way, algorithms such as RRT*smart, informed-RRT*, and RRT*FND relied on random sampling and struggled to find the optimal path. The RRT*Smart-AD algorithm, on the other hand, demonstrated a clearer goal-directedness and reduced the number of redundant nodes. By using the dynamic A* heuristic function and intelligent beacon sampling strategy, the overall path cost was further minimized, and the time required to reach the goal point was reduced. According to Table 3, in the 50 experiments conducted, the RRT*Smart-AD algorithm exhibited a better average path length and runtime compared with the other algorithms.

**Figure 14:**
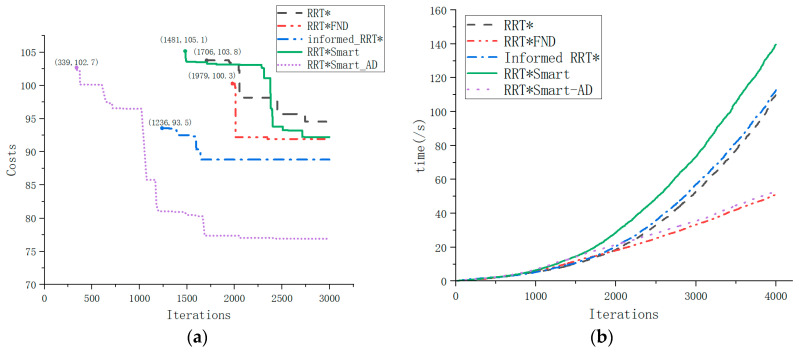
Comparative performance analysis of each algorithm. (**a**) Comparison of Algorithms Iteration-Cost. (**b**) Comparison of Algorithms Iteration-time.

The time recorded in Table 3 includes both the planning time and angle distance cost. Compared with RRT*Smart-AD, our method had a shorter planning time and produced significantly better sampling nodes and random tree scales. Figure 14 shows the iterative process and running time relationships of the path solution parameters for all asymptotically optimal algorithms investigated in this study.

From Figure 14a, we found the initial path to calculate the path costs. It can be observed that the RRT*Smart-AD algorithm quickly found an efficient path and optimized it. The RRT*Smart-AD algorithm outperformed the other algorithms in terms of the number of iterations, search duration, and path distance. Due to the improvement in the dynamic A* random sequence sampling method, the RRT*Smart-AD algorithm could quickly find a relatively optimal path within around 500 iterations.

From Figure 14b, it can be observed that the FN-based RRT*FN algorithms, which involve node pruning, exhibited a linearly increasing trend in iteration time after a certain number of nodes, while other algorithms showed a concave increasing trend. The dynamic cost-to-come coefficient in the A* evaluation function, coupled with the intelligent beacon sampling used in the RRT*Smart-AD algorithm, further reduced the overall path cost and facilitated faster pathfinding.

### 4.2. Static Simulation Experiment of the Robotic Arm

To demonstrate the efficiency of the RRT*Smart-AD algorithm, static obstacle avoidance experiments were conducted using robotic arm 2 as a stationary obstacle. The robotic arm’s joint configuration space was considered to be a six-dimensional vector, denoted as $\left[\theta_{1},\theta_{2},\theta_{3},\theta_{4},\theta_{5},\theta_{6}\right]$. The mapping between the joint configuration space and the workspace was achieved through kinematic forward and inverse solutions. The RRT*Smart-AD algorithm was applied to find paths within the six joint configuration spaces.

For comparison, the RRT*Smart-AD algorithm was compared against the goal-biased RRT* and RRT*Smart algorithm. The initial joint poses and target joint poses for robotic arm 1 were as follows:

$$x_{start}=\left[-65{}^{\circ},-90{}^{\circ},-60{}^{\circ},0,0,0\right],\;x_{goal}=\left[60{}^{\circ},10{}^{\circ},-10{}^{\circ},60{}^{\circ},60{}^{\circ},120{}^{\circ}\right].$$

For all the compared algorithms, the parameters remained consistent. The step size was set to 3°, and the goal bias was set to 0.18. The population size for the PSO optimization was the number of initial path discrete points, and the number of iterations was 30. The joint poses for the stationary robotic arm were as follows:

$$x_{\mathrm{Static}}=\left[20{}^{\circ},45{}^{\circ},-30{}^{\circ},-60{}^{\circ},-60{}^{\circ},-60{}^{\circ}\right]$$

**Figure 15:**
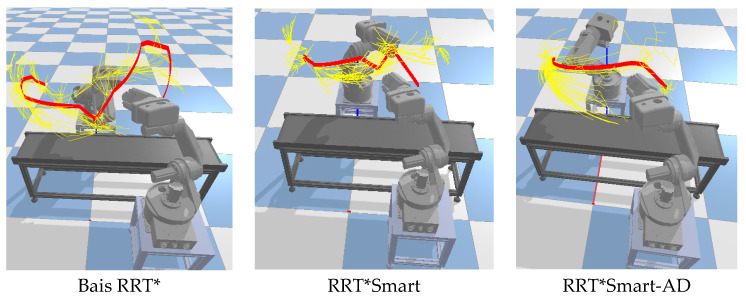
Robotic arm static path planning process.

In the planning path process results of the robotic arm shown in Figure 15, the yellow paths represent the sampled nodes, while the red path represents the final path. It can be observed that compared with the biased RRT* and RRT*Smart algorithms, the RRT*Smart-AD algorithm generated shorter and smoother paths. Additionally, the number of unnecessary search nodes was reduced, leading to an increased tendency toward the goal.

**Figure 16:**
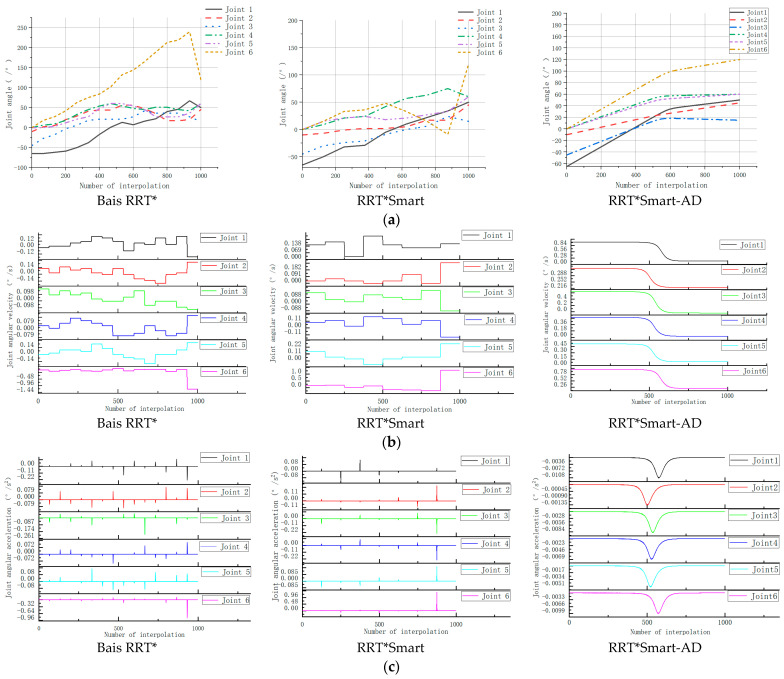
Comparison of joint angle, joint angular velocity, and joint angular acceleration. (**a**) Comparison of joint angle; (**b**) Joint angular velocity comparison; (**c**) Comparison of joint angular acceleration.

In Figure 16, during the motion process of the path generated by the RRT*Smart-AD algorithm, there are abrupt changes in the paths of goal-biased RRT* and RRT*Smart. However, when using the joint angles, joint angular velocities, and joint angular accelerations obtained by applying the PSO combined with quintic NURBS curve fittings, the robotic arm exhibited smooth and uninterrupted motion. According to Table 4, RRT*Smart-AD improves search efficiency and reduces Cost.

### 4.3. Dual-Robotic-Arm Collaborative Simulation Experiment

To demonstrate the reliability of this method, a dual-robot collaborative experiment was designed. The initial path was generated using the RRT*Smart-AD algorithm. During the tracking process, a dynamic region-based collision detection method was employed. Specifically, as each robot arm moved to an interpolated point, the model was used to predict and assess whether there would be a collision between the interpolated points of the two robot arms within a certain future time range. If a collision was predicted, the RRT*Smart-AD algorithm was used to repair or regrow the path during the robot arm operation to generate a valid path.

The initial joint poses and target joint poses of the main robot arm 1 were as follows:

$$x_{start}=\left[-33.0{}^{\circ}-43.5{}^{\circ}\;10.0{}^{\circ}\;0.0{}^{\circ}\;0.0{}^{\circ}-40.0{}^{\circ}\right],\;x_{goal}=\left[60.0{}^{\circ},59.0{}^{\circ}\;,50.0{}^{\circ},\;60.0{}^{\circ},\;60.0{}^{\circ},\;60.0{}^{\circ}\right].$$

The parameters of all the compared algorithms were kept the same, with a step size of 3° and an objective bias of 0.2, and the PSO algorithm performed the optimization with a population size of the number of discrete path nodes, with a number of iterations of 30, and from the joint position of the robotic arm 2 as follows:

$$x_{start}=\left[-33.0{}^{\circ},59.0{}^{\circ},10.0{}^{\circ},0.0{}^{\circ},0.0{}^{\circ},0.0{}^{\circ}\right],\;x_{goal}=\left[55.0{}^{\circ},59.0{}^{\circ},40.0{}^{\circ},0.0{}^{\circ},0.0{}^{\circ},0.0{}^{\circ}\right].$$

**Figure 17:**
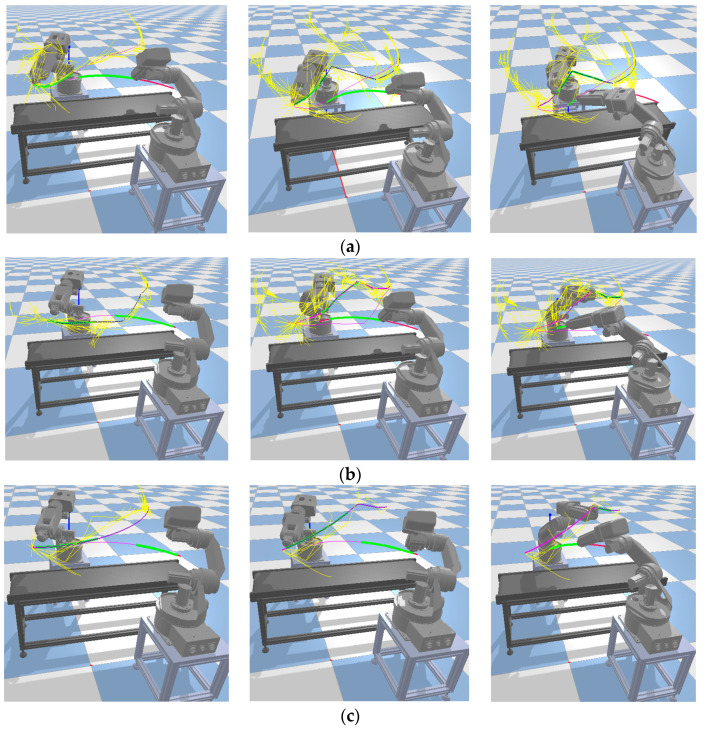
Comparison of dynamic path planning algorithms for robotic arms. (**a**) Bais RRT*. (**b**) RRT*Smart. (**c**) RRT*Smart-AD.

During the algorithm planning process, as shown in Figure 17, dual-arm cooperation interfered with the initially planned path. In Figure 17, two robotic arms are engaged in collaborative planning. The conveyor belt in the middle serves as an obstacle. The yellow path represents the sampled path, the green line segments indicate the dynamic detection area, and the purple path represents the motion trajectory. Both RRT* and RRT*Smart abandoned all path nodes after the collision point in the original path and planned to directly reach the goal. Because these two plans were based on RRT, they were not only time-consuming but also tortuous. The search algorithm used in the path repair and regeneration process of RRT*Smart-AD could quickly generate valid new paths, making the path more stable and sometimes more effective. Compared with RRT* and RRT*Smart, the generated sample points occupied more space. According to Table 5, across 50 simulation experiments, RRT*Smart-AD demonstrated better performance in terms of time and path cost. The generated initial discrete point paths were smoothed using quintic NURBSs, and the joint PSO performed robotic arm optimization trajectories on the generated quintic NURBSs. Dynamic-area path repair and regrowth were used to be more adaptive to dynamic obstacle environments.

**Figure 18:**
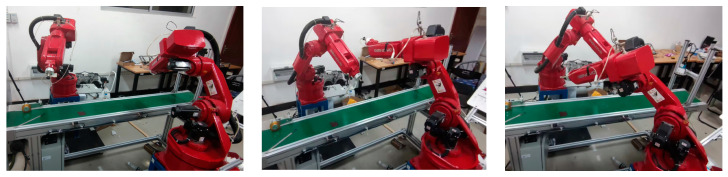
Dual-robotic-arm physics demonstration experiment.

The experimental data in Figure 17 was used for a dual robotic arm prototype demonstration, which showed the accuracy and effectiveness of the algorithm, as shown in Figure 18.

## 5. Conclusions

The proposed RRT*Smart-AD motion planner aims to address the cooperative dynamic motion planning problem of dual manipulator arms. This method was designed for six-DOF dual-arms cooperation in complex scenarios, to obtain cooperative motion trajectories that adhere to joint ranges, differential constraints, and physical collision constraints. The effectiveness of the algorithm was validated through simulation comparisons and real robot experiments. These experiments demonstrated that the robotic arm could effectively navigate static global obstacles and execute dynamic dual-robot cooperative trajectory planning, showcasing a motion process characterized by exceptional smoothness, intelligence, and flexibility.

Previous research failed to address the cooperative motion planning of dual manipulator arms in terms of sampling methods, path optimization, and dynamic adaptability. The proposed RRT*Smart-AD motion planner addresses these issues. In the motion planner, to enhance efficiency, a dynamic A* cost function sampling method is introduced in combination with an intelligent beacon sampling method. Compared with traditional methods, like random sampling and goal-biased sampling in RRT, this approach significantly accelerates the discovery of effective paths, thereby improving the exploration speed and efficiency. Additionally, dynamic regional path repair and regrowth strategies are proposed to enhance adaptability in dynamic scenarios. These strategies prevent the discarding of original path nodes and enable the rapid generation of new paths within dynamic environments. PSO optimizes the motion trajectory by optimizing the parameters of the quintuple NURBSs for path-smoothing interpolation to avoid collision problems. The use of higher-order spline functions ensures the continuity of acceleration and bumps during the smoothing process.

However, there are certain limitations to this approach. First, in scenarios involving dual manipulators working together, the manipulators are categorized as primary and secondary. Although the obtained results generate optimal trajectories for the primary manipulator, the motion trajectory of the secondary arm is catered to the motion trajectory of the primary arm by sacrificing the optimality of the secondary arm. In future research, it would be worthwhile to explore strategies for achieving equal optimization of both manipulators, such as equipartition and mutual avoidance in dual manipulator collaboration. Second, the use of maximum curvature could be further investigated in a wider range of trajectory optimization applications to ensure the generality of PSO in conjunction with the quintic NURBSs optimization method.

## Figures and Tables

**Table 1:** Comparison of advantages and disadvantages of path planning methods.

Algorithms	Advantages	Disadvantages
Genetic algorithms [4]	Parallel processing, global search capability, and problem domain flexibility	Easy to fall into local optimality, long running time, slow convergence, many sensitive parameters, and poor adaptation to dynamic environment
Particle swarm algorithms [5]	Parallel processing, strong global search capability, adaptive search strategy, and adaptive to problem constraints	Multiple and sensitive parameters, easy to fall into local optimal solutions, long running time, and poor adaptability to dynamic environments
Ant colony algorithms [6]	Parallel processing, dynamic environment adaptability, and strong global and local search paths for large-scale problems	Long-running time, poor convergence, many sensitive parameters, poor solution of high-dimensional complex problems, easy to fall into local optimization, and the contradiction between population diversity and convergence
A* algorithm [7]	Exploring spatial completeness, optimality, and flexibility in heuristic function sampling	Long running time, large number of node extensions, slow convergence, poor adaptation to dynamic environments, poor solvability of high-dimensional complex problems, and poor global search capability
APF algorithm [8]	Local path optimization, dynamic environment adaptability, and high operational efficiency	Easy to fall into local optimization, sensitive to parameters such as repulsive and gravitational coefficients, poor solvability for high dimensional complex problems, and limited scalability
RRT [9]	Efficiency in high-dimensional spaces, probabilistic completeness, strong global search capability, and adaptability to complex environments	Suboptimality, poor adaptation to dynamic environments, presence of redundant nodes, sampling node redundancy, and insufficient regional exploration
RRT* [10]	Efficiency in high-dimensional spaces, strong global search capability, adaptation to complex environments, and asymptotically optimal solution	Poor dynamic environment adaptability, low path optimization efficiency and long running time, sampling node redundancy, and insufficient regional exploration
RRT*Smart [28]	High operation efficiency, strong global search ability, strong local optimization ability, adapt to complex environments, asymptotically optimal solution, and high efficiency of path optimization with the proposed intelligent sampling method	Poor dynamic environment adaptability, long consumption time, sampling node redundancy, and insufficient regional exploration
Improved informed-RRT* [11]	Application for robotic arms and heuristic elliptic sampling to obtain global near-optimal solutions	Because the heuristic sampling method samples in a three-dimensional ellipse, each sample needs to inverse the position of the end of the robotic arm; the actual operating efficiency is low; the expandability is extremely poor, and thus, it is not possible to expand the flexibility of the joints of the robotic arm; energy optimization; the dynamic environment is poorly adapted; the operating efficiency is low; and so on
Improved RRT* [12]	Application to the W-space of a robotic arm to add target bias sampling methods	Poor adaptability to dynamic environment, low path optimization efficiency and long consumption time, easy to fall into local optimum, and low operation efficiency
Improved RRT*FN [13]	Application for robotic arms, global approximate optimal solution, increasing the number of fixed nodes strategy exploration efficiency is further improved, and dynamic environment adaptability	Overly biased sampling and inefficient path optimization, easy to fall into local optimum, and low operation efficiency
P_RRT* [14]	Applications to robotic arms, global approximate optimal solutions, partial strategies for fusing APFs, matrix and target deviation sampling guidance, and dynamic environment adaptation	Easy to fall into local optimum, more sensitive parameters, low efficiency of path optimization, and poor scalability
Rt-RRT* [15]	The proposed online reconnection strategy is adaptable to dynamic environments, applicable to high-dimensional space, and has high operational efficiency	Sampling bias, inefficient path optimization, easy to fall into local optima, sampling node redundancy, insufficient regional exploration, and no application of robotic arms
RRT*FND [16]	Applied to robotic arms and the proposed reconnection strategy and re-growth strategy are highly adaptable to dynamic environments	Path optimization is inefficient and easily falls into the local optimum; the reconnection strategy can quickly generate effective paths in dynamic environments, but the probability is not optimal paths; sampling node redundancy; and insufficient regional exploration
GA-RRT [17]	GA-RRT adds target bias and a new sampling method to improve the speed of generating effective paths for dual-arm motion planning	Non-optimal paths, prone to getting stuck in local optima, and the effectiveness of the path optimization method is average
VT-RRT [18]	The improvement of dynamic step size and target increases the efficiency of generating paths in complex environments to cope with dual-arm motion planning	Non-optimal paths, prone to getting stuck in local optima, and the effectiveness of the path optimization method is average
Spline-RRT* [19]	Spline has a good optimization effect on the motion trajectory of the manipulator arm	The running time is too long, prone to getting stuck in local optima, and not suitable for complex scenes
Improved RRT* [20]	Exploration efficiency is high for the four-tree algorithm, which generates paths quickly; adding potential energy enables the generation of optimal paths in local space	The generated paths may not be optimal, the optimization algorithm’s effect on the motion of the manipulator arm is average, and it is prone to getting stuck in local optima and may fail to find an effective path
[21]	Applying the fast execution efficiency of RRT-Connect, it can be used for path planning of dual robotic arms; this is a simple application of the RRT-Connect algorithm	Non-optimal paths, prone to getting stuck in local optima, and the effectiveness of the path optimization method is average
[22]	Applying the fast execution efficiency of RRT-Connect, it can be used for path planning of dual robotic arms	Non-optimal paths, are prone to getting stuck in local optima, and the effectiveness of the path optimization method is average

**Table 2:** Nominal link parameters of the robot.

link*_i_*	*θ_i_*/°	*α_i_*/°	a*_i_* (mm)	d*_i_* (mm)	Range of (*θ_i_*/°)	Maximum Velocity (°/s)
1	$\theta_{1}$	90	110	320	−170~170	180
2	$\theta_{2}+$90	0	290	0	−45~135	180
3	$\theta_{3}$	90	1210	0	−70~130	225
4	$\theta_{4}$	90	0	310	−170~170	300
5	$\theta_{5}$	90	0	0	−120~120	375
6	$\theta_{6}$	0	0	111.5	−360~360	500

**Table 3:** Experimental results of static path planning in two-dimensional space.

Algorithms	Cost (mm)	Time (s)
Bias RRT*	1231.48	80.97
RRT*Smart	1226.88	86.98
Inform RRT*	1201.87	91.19
RRT*FN	1282.98	46.34
FMT*	1194.27	52.84
RRT*Smart-AD	790.22	44.68

**Table 4:** Comparison of static path planning results of the robotic arm.

Algorithms	Bias RRT*	RRT*Smart	RRT*Smart-AD
Cost (°)	1466.59	1230.75	1177.95
Time (s)	510.38	530.39	186.16

**Table 5:** Comparison of path planning results of the robotic arm.

Algorithms	Bias RRT*	RRT*Smart	RRT*Smart-AD
Cost (°)	1582.66	1534.37	1272.19
Time (s)	669.64	761.18	203.61

## Data Availability

Data sharing not applicable. No new data were created or analyzed in this study. Data sharing is not applicable to this article.
